# Understanding Factors Motivating Trainees to Pursue a Career in Radiology: An Interview Study

**DOI:** 10.7759/cureus.102745

**Published:** 2026-01-31

**Authors:** Rebekah Brawley, Sanjana Kumar, Jonathan Kibble

**Affiliations:** 1 Medicine, University of Central Florida College of Medicine, Orlando, USA

**Keywords:** medical education, medical student specialty choice, motivation factors, radiology medical education, specialty choice influencers

## Abstract

This cross-sectional qualitative study investigated the factors that motivate trainees to pursue diagnostic radiology and examined how perceptions of the specialty may evolve across different stages of medical training. Semi-structured interviews were conducted with a purposive sample of three medical students, three PGY-5 diagnostic radiology residents, and four board-certified radiologists. Thematic analysis was applied to the transcripts to identify recurring themes and subthemes across participant groups. Across all training levels, participants consistently cited the intellectual rigor of radiology and a strong passion for lifelong learning as key motivators. The interdisciplinary nature of the field and its central role in patient care were highly valued. Interviewees emphasized the importance of early exposure, mentorship, and addressing persistent misconceptions to increase interest in radiology. The decision to pursue the specialty was described as a reflective process involving alignment with personal values, strengths, and career goals. Choosing radiology requires introspection and a clear understanding of one’s professional identity and aspirations. While individual journeys vary, shared themes suggest that targeted interventions, particularly early curricular integration and accessible mentorship, may foster interest and reduce barriers to entry. These findings offer actionable insights for medical educators seeking to support students in exploring radiology as a viable and fulfilling career.

## Introduction

Uncertainty about specialty choice is a persistent source of stress for many medical students. Analysis of the AAMC Year 2 and Graduation Questionnaires (2016-2020) revealed that 56% of students changed their intended specialty between the end of the second year and residency application [1]. This highlights the third and fourth years in general as a critical period for solidifying career decisions, likely due to increased clinical exposure. Radiology, in particular, sees significant shifts in student interest during medical school. According to the NRMP’s Charting Outcomes in the Match report for 2024, 899 MD students applied for diagnostic radiology, 4.7% of all MD applicants across 22 specialties [2]. Yet only 1.8% of this cohort had identified radiology as their intended specialty upon matriculation, based on the AAMC’s Matriculating Student Questionnaire [3]. This suggests that many students discover radiology during their training. Further supporting this trend, radiology ranked second, after Physical Medicine and Rehabilitation, for attracting the highest proportion of students who were undecided at matriculation, according to the AAMC’s report on entering residents [4]. Compared to other specialties, the medical school experience appears particularly influential in guiding students toward a career in radiology.

Exploring the factors that influence the choice of diagnostic radiology as a specialty is, therefore, a compelling area of research. The factors motivating students to select radiology as a specialty choice have been partially described in earlier studies using surveys [5-8]. Medical students may be discovering radiology almost despite the formal curriculum, since previous reports have suggested they often receive limited formal exposure to radiology during their training. Furthermore, this lack of exposure can lead to a limited understanding of the field and the persistence of negative stereotypes, such as radiologists being antisocial, working long hours in dark rooms, and having minimal patient interaction [9]. These misconceptions may discourage more students from considering radiology.

Given that medical students may have certain misconceptions about the field of radiology [10], it is important to assess how, or if, the experience of being a radiology trainee aligns with perceptions and expectations one holds of the specialty as a medical student. In one study evaluating resident physicians’ perceptions of diagnostic radiology, most radiology residents disagreed that radiology is monotonous, and a majority felt that radiologists see most of the interesting cases in the hospital, whereas most non-radiology residents took on opposing views [7].

Previous studies have used surveys to assess the role of a variety of factors in motivating students to pursue radiology [5-8]. Yet, few studies, if any, have used interviews as an approach to gain the personal insight and reflection of trainees regarding their specialty choice. Our study employed a cross-sectional approach through interviews of third- and fourth-year medical students, PGY 2-5 radiology residents, and board-certified radiologists. The main objectives of this exploratory study were to (i) identify factors motivating the choice of radiology as a specialty, (ii) assess how perceptions of radiology shift across training levels, and (iii) identify opportunities to enhance radiology advising and mentorship for students as they explore radiology as a potential career path. Secondly, we aimed to use the cross-sectional nature of the study to assess how motivational factors change across levels of training. Finally, this study sought to identify how we can improve students' understanding of radiology and eliminate the barriers students face in considering radiology as a specialty through insights from current radiologists, radiology trainees, and medical students.

## Materials and methods

Research design

A qualitative research methodology using semi-structured interviews was used to address the research questions. The approach used principles from interpretive phenomenological analysis (IPA). IPA studies allow for a detailed analysis of the experiences of each individual, while also identifying general themes across subjects [11]. The research team was composed of two medical students in their preclinical training years and a faculty student affairs dean. The student researchers interviewed all participants. 

Participants and recruiting

IPA studies typically use purposive sampling to recruit a relatively homogeneous group of participants who share a common lived experience, in this case, pursuing a career in radiology. To introduce a limited degree of heterogeneity, we deliberately sampled participants across different stages of professional experience, from trainees to established radiologists. This cross‑sectional approach was intended to capture a range of perspectives and to explore whether and how meanings and experiences shift across career stages. Medical students from the third and fourth years of the M.D. program at a Southern U.S. medical school, PGY 2-5 radiology trainees who graduated from the school, and board-certified radiologists from the surrounding geographical area were invited to participate in the study. Sample sizes in IPA studies typically range from 1 to 12 participants [12]. Recruitment emails were sent to participants via the College of Medicine Office of Student Affairs. The email invitation provided participants with access to the explanation of research and contact information of investigators to schedule an online interview at a time and place of their convenience. Participation in the interview was voluntary, and no incentive for participation was offered. 

There are approximately 120 students per year in the M.D. program at UCF COM. Recruitment yielded three medical student participants, including one M3 and two M4s. Out of 28 UCF COM graduates currently in radiology residency training, we were able to contact 17 residents. Three PGY-5 diagnostic radiology residents agreed to participate in the study. Out of 107 radiologists from UCF COM’s volunteer faculty list, four board-certified radiologists participated in the interview process. The radiologists had been practicing radiology for approximately 20 to 32 years. A total of 10 interviews were conducted. 

Data collection and analysis

Semi-structured interviews were conducted over virtual Zoom calls, lasting ~30 minutes each. Questions were structured and presented in a manner that allowed interviewees to explore their experiences in depth. The interview guides are shown in Table 1. Follow-up questions were used as needed to ensure each question was properly addressed. Each interview was recorded and transcribed verbatim for analysis. The coding process in IPA differs from conventional thematic analysis. Each transcript is analyzed individually, with initial open, exploratory notes made directly on the text to support meaning‑making rather than categorization. Coding is interpretive rather than procedural, and this first analytic pass allows emergent themes to develop within each case. As additional cases are analyzed, a cross‑case analysis is undertaken in which patterns of convergence and divergence are examined, and some themes become more robust. Through subsequent iterative readings of the data, focusing on connections across exploratory notes and emergent themes, higher‑order themes are ultimately developed. 

**Table 1:** Interview Guide

Question Category	Interview Question
Background Information	For M3/M4: Please state which medical school class you are in. For radiology residents: Please state your PGY year. For board-certified radiologists: Please state how many years you have been practicing.
Motivating Factors	What sparked your interest in radiology? What continues to motivate you to pursue radiology? What are the qualities that make a successful radiologist, and what qualities do you have that make radiology a good fit for you as a specialty?
Experiences and Expectations	What about radiology brings you a sense of fulfillment? [For M3/M4: How do you anticipate radiology will bring you a sense of fulfillment?] Has the experience of being a radiology trainee/radiologist met your expectations? [For M3/M4: What are some expectations you currently have for being a radiology trainee?] Have your perceptions of radiology changed as you’ve progressed throughout your training?
Reflections	Lastly, I’d like you to think about your journey throughout medical school and training thus far: As you reflect on your journey, was there any point at which you would have liked more support in helping you decide which specialty to pursue, particularly in choosing radiology? How can we improve radiology education/exposure for medical students, or best guide students interested in radiology? Are there any other comments you have on radiology, or pursuing radiology that we have not covered?

Conforming to IPA norms, the two student researchers each individually analyzed all transcripts, making their own notes and identifying their initial themes and subthemes across participants. Our quality assurance process involved initial meetings where the student investigators shared their primary analyses and engaged in further interpretive discussions to reach consensus about themes. The documents were shared among the whole research team and were further interrogated by the faculty author to reach consensus on the final themes presented. Since IPA does not develop a conventional codebook used in other kinds of thematic analysis, it was not possible to calculate interrater reliability metrics. The study received approval with Exempt status from our institutional review board. 

## Results

Medical students

Medical Student Interviews: Motivation to Pursue Radiology

For medical students, experiences in the clinical years were vital in sparking interest in radiology. While one student entered medical school with an initial interest in radiology, the others discovered the specialty through progressive exposure. Clinical rotations provided a dual function: they helped students rule out previously considered specialties while simultaneously allowing students to gain a greater understanding of the role of imaging in patient care. Students consistently remarked on the fact that imaging intersects with nearly every specialty, which expanded their appreciation for radiology’s central role in medicine. 

“I think mostly being on clinical rounds when you realize that every specialty still needs imaging… And I've really enjoyed that so much more than I thought I would, looking at the images myself and then comparing them with the radiologist's notes.”

Students anticipated finding professional fulfillment in diagnostic accuracy and problem-solving. They described radiology as intellectually stimulating, with the opportunity to synthesize knowledge across organ systems:

“I admire the breadth of knowledge that's required to be a radiologist. You have to know anatomy and pathology from every body system, and location, and so that desire to learn all of that continues to motivate me.”

Background experience also appeared key in helping students construct a sense of fit with radiology. Personal interests in physics and prior training in detail-oriented fields gave students confidence that radiology matched their innate strengths. One student believed her background in literature editing helped her develop a fine attention to detail that will be useful in radiology: 

“…It's just you have to have the eyes for it. You have to have the patience and kind of have a structure to how you approach looking at an image. You need to be systematic in your approach with all images, and you need to be detail-oriented.”

Medical Student Interviews: Expectations for Radiology Training

Expectations were shaped by both initial misconceptions and progressive corrections. All students’ perceptions of radiology greatly changed as they progressed throughout medical school. Initial assumptions that radiologists lacked patient interaction gave way to the recognition that subspecialties involve substantial patient contact:

“For one thing, I really didn't realize that radiology, depending on the subspecialty you go into, you can actually have quite a bit of patient encounter, if that's what you want. And for me, I'm especially interested in breast radiology or radiation oncology and I think my perception prior to knowing more about the field was that it was just some stranger sitting in a dark room reading images, and I thought how lonely that has to be.”

Expectations for radiology training centered around the learning environment and demands of training. One medical student focused on the variety of cases she would like to have exposure to, and the teaching style she would like to see embodied by her attendings. Another student expected radiology residency to be highly demanding, requiring significant studying: 

“Radiology residency is going to be a lot of work, mostly because it's a lot of self-study. I'm not in the hospital the whole time. But I'm constantly studying and learning medicine and radiology throughout that whole period.”

Medical Student Interviews: Reflection

As previously noted, most of the medical students did not ultimately decide to pursue radiology until their clinical years of medical school. While one student believed naturally going through the process of exploring specialties led her to radiology, all students expressed a desire for greater faculty support in helping decide which specialty to pursue. One student cited an institution-specific lack of a faculty diagnostic radiologist as a challenge:

“I would have liked more support in terms of having access to a diagnostic radiologist, like on - faculty. The closest connection [I had] to diagnostic radiology is, in fact, an interventional radiologist. And so, because of that, I kind of feel like I didn't get as much exposure to diagnostics as I would have liked, or opportunities to pursue diagnostic radiology research as I would have liked.”

One student highlighted the critical role of community in fostering sustained engagement with radiology. While formal student interest groups serve as initial points of contact, they are often limited in time and capacity. With ideas such as group chats for sharing opportunities and advice, this interviewee underscored how informal, peer-driven communities can help promote a sense of shared purpose and collective momentum among students pursuing the specialty. Interestingly, this student also emphasized the importance of individual initiative. While institutional support and community connections were seen as valuable to this student, the interviewee believed that students must take proactive steps to find opportunities for themselves. 

All students also suggested earlier and more exposure to imaging in the medical school curriculum as a way to improve radiology education in medical school:

“But I wish more towards 3rd or at some point throughout third year, we had something more dedicated, maybe like a short two-week rotation where we could rotate with some radiologists to sort of get that feel.”

Due to lack of exposure to radiology, students discussed how misconceptions perpetuate among medical students, and the importance of improving understanding of the field:

“And I think it's much more involved in medicine than I think most people understand… And it's not somebody sitting in a dark room looking at pictures. And I think that's the reputation it has. And there's like diagnostic, there's interventional- it's become a huge field. So, I think it's really important, and I wish it would be more emphasized.”

Radiology residents

Resident Interviews: Motivation to Pursue Radiology

For radiology residents, third- and fourth-year clinical rotations were also a key factor that led them to radiology. Two of the residents had planned on pursuing different specialties and did not ultimately decide to pursue radiology until their fourth year of medical school. Peer influence and informal conversations also played a meaningful role in shifting career direction: 

“…there was a TY student who was going into radiology, and I was talking to him about my, you know, dilemma and specialty choosing. I mentioned my interest in ortho and I don't know what else I might have said, but you know, I guess I didn't bring up radiology. He's like, oh, ever hear of MSK or musculoskeletal radiology? And I was like no, I never heard of that, never even thought of radiology. And then pretty much started looking into it from there.”

Motivation behind pursuing radiology centered around factors including the intellectual challenge, feelings of personal competency, being the ‘doctor’s doctor’, and driving patient care. Residents expressed a commitment and appreciation to the learning process and expressed finding fulfillment and motivation from achieving diagnostic excellence in a manner that benefits patients. Furthermore, residents also found satisfaction in the ability to be of value to other specialists:

“I really enjoy how they call radiology is like being the 'doctor's doctor.' You're talking a lot with your colleagues and making plans and you know, helping really drive clinical care.”

Residents outlined personal qualities they thought aligned well with being in radiology, including being self-driven and efficient, cool under pressure, and a team player. Residents also identified personal alignment with radiology’s unique professional culture. One resident appreciated the field’s relative emotional distance compared to specialties with high volumes of direct patient care. As radiologists are not always with patients at the bedside, this resident described the importance of being okay with not getting credit. On the flip side, other residents valued the collaborative, consultative role of radiology. 

Resident Interviews: Alignment of Expectation and Reality

When considering how radiology residency aligned with expectations coming into training, residents had difficulty articulating pre-residency expectations, largely due to poor exposure during medical school. Nevertheless, both residents described developing a greater appreciation for radiology’s overall role in the medical field throughout their training: 

“I think you definitely become more aware of how important the radiologist is in the medical team and in the medical system in general. I don't think I realized how crucial medical imaging was in the medical field and how utilized it was even if over utilized.”

Residents reported both positive experiences (social resident culture, meaningful collaboration with other physicians) and challenges of residency (insufficient faculty teaching due to workload, high self-study demands). Artificial intelligence (AI) emerged as a recurrent theme. Residents maintained a pragmatic outlook. While acknowledging that AI will influence the field, one resident expressed confidence in radiology’s resilience due to the complexity of interpretation and clinical integration required: 

“And you know I certainly questioned when I was a med student, and when I was an intern when someone would say like, oh, your job's going to be taken over by AI, and now I'm on this end and I'm like, okay, good luck to AI with dealing with some of the stuff that we deal with.”

Resident Interviews: Reflection

Each resident offered a different perspective pertaining to the level of support they would’ve liked in helping them pursue radiology. While one resident expressed that the institution-specific lack of a faculty diagnostic radiologist was challenging, another resident described how personal initiative helped him find the support he needed in pursuing radiology: 

“I mean a lot of people, even in my residency- the way most people find out is either they had a family member in radiology, or they just really didn't find anything in their rotations in med school that they really enjoyed. And then they kind of stumbled across radiology and found it that way. And that's kind of how I did it. And then, once that happened I just kind of reached out on my own to local radiologists, people that [I] was kind of tied in with.”

Despite this, residents underscored the importance of enhancing radiology exposure in the medical school curriculum and particularly providing earlier exposure:

“I think medical students certainly need more exposure. I think, like I said, radiology can have a PR problem sometimes. And people don't realize that it's not just about sitting in a dark room and looking at pictures all day long.”

Residents valued having radiologists on medical school faculty to enhance radiology curricula and promote direct interaction with those in the field, with one resident saying:

“A more robust exposure to having some kind of structured radiology curriculum. I don't know how the curriculum has changed, but we used to have the longitudinal themes, or whatever they were. I can't remember having radiology included in that. And if I remember correctly, some of our small group cases and that kind of thing had had radiology in it. But, having a radiologist present to provide that perspective, I think, is equally as important as including it in the curriculum itself. And then including radiologists on faculty.”

One resident provided an example of how his current institution is taking steps to incorporate radiology into their medical school curriculum: 

“But here at -, I give lectures to the med students, and they sort of have a more dedicated, yearly sort of, built-in radiology curriculum, where we give sort of like, just lectures. For example, next week I'm giving an Intro to radiology lecture. Towards January, they have, like an MSK radiology lecture. So, they have that sort of built into their curriculum. So, I think this starts during their second year. So, that's even earlier exposure.”

One resident discussed his desire for medical students' misconceptions of radiology to be addressed at length, and suggested radiology interest groups could be a vital tool in providing a better understanding of radiology: 

“Maybe taking advantage of radiology interest groups at medical schools and ensuring that you have sessions where radiologists just come in and either do procedures or simulations or take medical students through interesting cases or something, just to kind of get them interested in what radiologists do and try to provide that perspective that it's not just about reading images.”

Both residents commented on the current and future job market. While one resident noted the current high demand for radiologists, a different resident argued that the long-term impact of AI should be a factor when future medical students consider radiology. However, residents expressed an overall positive outlook on choosing the field despite the prevalence of conversations surrounding potential infiltration by AI: 

“But overall, it's an incredibly fascinating field. And even just talking about AI, we're talking about it because radiology has the most advanced technical kind of gear and products out there. And it's an ever-changing field. And I think that's also a reason to be in it if you want to be a part of something like that, so it's definitely the best specialty still. And I wouldn't have chosen anything else.”

Rather than major concerns for AI, the residents had some more prosaic workplace concerns, first about disproportionately rising clinical volumes negatively impacting educational time: 

“But there's been a really drastic rise in clinical volumes and I think radiology has felt it. There are more non-physician providers who are ordering imaging studies a lot of times to kind of compensate for maybe not having as much experience. And so I think imaging volumes have risen even more than just the clinical volumes. And so we're in this weird space in radiology where we just don't have enough people. And there's really no ability to utilize a physician extender, or whatever you want to call them, like NPs and PAs, for example in diagnostic radiology… But anyways, with that, what's happened in residency is as academic centers get busier, and as our faculty get busier, education tends to take the back burner, and so I felt myself throughout residency craving a little bit more teaching, a little bit more learning, and I wasn't anticipating having to do so much on my own. So, that's been a little bit unfortunate.”

An additional concern in the post-COVID workplace was the negative impact of increased remote working among radiologists: 

“..radiology is in a unique position within medicine to effectively, I don't know if outsource is the right word, but we're having more and more remote radiologists. In fact, we have several remote faculty at my institution. And I like all of them. They're great. They teach really well, they're excellent radiologists. But, they found a position that works for them where they get to do their job from home. And so there's been a lot of conversation within radiology about the value that we provide, the importance of being in person, the importance of being on site in hospitals. Interestingly enough, both at my institution and other institutions, radiology is kind of continually being kind of pushed out of hospital spaces and into like the periphery.”

Radiologists 

Radiologist Interviews: Motivation to Pursue Radiology

Practicing radiologists reflected on diverse paths into the specialty. For some, a fourth-year elective shifted their trajectory away from other fields, while others cited formative early life exposures and background experiences. For example, one radiologist gained exposure to imaging departments at a young age as her mother was a sonographer. For another radiologist, his love for geography and visual learning led to his affinity for radiology: 

“I was a geography nerd when I was a kid, and anatomy is just geography of the body. And so, I immediately took to it, because it was just like looking at maps…”

As similarly noted by radiology residents, radiologists found motivation and fulfillment from being able to impact patient care. Radiologists took pride in achieving diagnostic excellence for their patients while helping a variety of other doctors involved in patient care. All expressed the importance of being patient-oriented and approaching their work with care. Radiologists also valued the intellectual challenge of radiology, admiring the amount of knowledge required to solve complex patient cases. A sense of curiosity, ability to work fast, adaptability, and dedication to continuous learning and growth were all examples of important qualities for radiologists. Having great attention to detail was also commonly discussed:

“And having the patience to look at the images and be critical of what you're seeing. Just always be on guard. There are certain things as a radiologist you're only going to see a few times in your career. And they're very unusual things. But if you miss them, they could potentially kill the patient.”

Regarding feelings of personal fit with radiology, the radiologists had varying degrees of comfort with patient contact. One radiologist in particular enjoyed the ability to directly interact with patients through interventional neuroradiology. Another radiologist, however, felt that radiology matched his desire for less direct patient contact: 

“I wasn't that terribly comfortable with patient contact and being that intimately responsible for the patient. So, radiology was a good way to certainly contribute to the patient's care without being the one who is responsible for them continuing to breathe directly.”

While the level of comfort with patient contact varied, the importance of being able to work well with others was a common theme. Describing this, one radiologist said: 

“I think you have to be the type of person that, despite the fact that people joke with us that we sit in our dark room, and we don't really interact with people, the truth of the matter is we interact with everyone. We interact with patients, we interact with radiology technologists, we interact with just about every single specialist across the spectrum. So, you really have to be able to communicate well.”

Radiologist Interviews: Alignment of Expectation and Reality

Several radiologists reflected on the rigor of radiology residency, describing it as more demanding than anticipated. One radiologist commented on the high quantity of required reading and fast-paced environment he was faced with when he entered the field:

“I thought it was going to be I'm going to sit in a dark office and with air conditioning and drink coffee and eat donuts and have my feet up, and just leisurely have a nice academic existence where it would be nice and slow paced. And that's not the way it was. My first shock was when I got to residency and was presented with stacks of textbooks.”

A common theme across radiologists was a feeling of overall satisfaction with their careers. Radiologists commented on a genuine love for the field, lack of regret, and one radiologist even expressed a sense of gratitude for the way it has provided for his family. Radiologists also noted the evolution of radiology over time, particularly the rise of sub-specialization and technological innovation, which they viewed as positive shifts toward greater work-life balance and improved patient care. An overall passion for the field was evident: 

“I still do about 0.2 of radiology. And I actually, now I look forward to my days on radiology like I count the days because it's really the days where I'm able to kind of, I don't want to say relax, but I'm able to let go of some of the administrative headaches that I'm constantly dealing with, and the fires that I'm always putting out and really just get back to focusing on where my original passion is.”

Radiologist Interviews: Reflection

The practicing radiologists offered differing perspectives regarding the adequate level of guidance to help one in the process of choosing a specialty. While two of the radiologists expressed that they would have liked more positive mentorship in their pursuit of radiology, the other two radiologists saw specialty choice as a deeply personal decision that requires one to understand the type of people one wants to be around, and the type of work that brings one a sense of happiness.

Radiologists discussed a variety of ways to improve medical students' exposure to radiology. Among them, one radiologist discussed the importance of radiologists having enhanced interaction with medical students, which she believes requires more support from medical schools themselves:

“I think if there were more mentors, more time for mentors to spend with mentees, that would be key. Unfortunately, we're just all like super busy and you know, there has to be a way that the medical school can help support mentors to have more time.”

Several radiologists also advocated for required radiology rotations regardless of the specialty medical students ultimately choose to pursue. In addition, there was also support among radiologists for earlier exposure in the pre-clinical years of medical school through curriculum integration: 

“I would start in the first year because radiology really is anatomy. You have to know your anatomy but being able to apply it to something practical. Like when you take neuroanatomy, they showed you all the circuits. I'd rather show you those same circuits or similar circuits on an MRI.”

Considering that perceptions of radiology from those not in the field vary, one radiologist discussed how early exposure is essential to provide medical students with a better understanding of radiology and consider it: 

“And I also think that there probably are medical students out there who just don't even realize that they would make great radiologists if they just understood what radiology is. And if you don't expose them to radiology, they never have that opportunity to even have a chance to understand, or to seek, or find that flame so to speak.”

Increasing the number of students pursuing radiology was a common theme. Radiologists also noted the growing demand for imaging, which has heightened the need for more trainees. They pointed to interventional radiology as an especially appealing hybrid pathway for future students, providing the ability to have more patient interaction and do procedures:

“The diagnostic side is beautiful. The interventional side is also beautiful. Put them both together. You got the best of two worlds in one. And you can have interaction with patients.”

On the topic of AI, radiologists offered an overwhelmingly optimistic outlook. They acknowledged uncertainty but saw AI as a tool that reinforces radiology’s role as a technologically advanced, ever-evolving specialty. One radiologist in particular provided a strong viewpoint on the impact of AI: 

“And I just want people that you know, students that you know, have an inkling for radiology not to run away from radiology because they think that radiologists aren't going to be needed in the future, like that's never going to happen.”

A tabulated summary of the main themes synthesizing insights from students, residents and faculty participants is provided in Table 2.

**Table 2:** Themes and Subthemes Across Interviewees

Domain 1: Motivating Factors	
Themes	Subthemes
Intellectual Curiosity & Challenge	Desire for diagnostic problem-solving; Breadth of knowledge across organ systems; Radiology as 'detective work'; Lifelong learning
Personal Fit & Competency	Attention to detail; Visual learning; Aligned backgrounds (e.g., in engineering, editing, physics); Innovative-minded; Adaptability; Team player
Versatility & Breadth	Exposure to all specialties; Fellowship opportunities; Subspecialty diversity
Impact on Patient Care	Central role in diagnosis; Driving clinical decisions; 'Doctor’s doctor' identity
Lifestyle & Work-Life Balance	Predictable hours; Flexibility; Job market strength
Technological Engagement	Interest in emerging tools, AI integration; Digital imaging systems
Domain 2: Alignment of Expectation and Reality	
Themes	Subthemes
Training Demands	Steep learning curve; Heavy self-study; Academic rigor
Misconceptions & Stereotypes	‘Sitting in a dark room’ myth; Lack of patient contact; Radiologists as antisocial.
Subspecialty Variation	Patient-facing roles (e.g., breast imaging, IR); Procedural opportunities; Interdisciplinary collaboration
AI & Future Outlook	AI as a tool, not a threat; Efficiency enhancer; Long-term uncertainty
Career Satisfaction	Fulfillment from diagnostic accuracy; Long-term career satisfaction; Financial and professional stability
Domain 3: Reflections	
Themes	Subthemes
Exposure & Timing	Late discovery of radiology; Lack of early curriculum integration; Need for structured rotations
Mentorship & Support	Difficulty finding mentors; Importance of informal networks; Faculty accessibility
Curricular Recommendations	Integration of imaging into preclinical education; Required radiology rotations; Radiologist-led sessions
Community & Peer Support	Informal group chats; Sharing opportunities; Building radiology interest group infrastructure
Identity & Professional Culture	Radiology as inclusive of diverse personalities; Value of humility and collaboration; Pride in being 'behind the scenes'

## Discussion

Before discussing our study objectives, it is important to acknowledge its limitations. Although we conducted an in-depth analysis of 10 interviews, the sample is limited to a single allopathic medical school in the Southern United States, which may impose a geographic limitation on the transferability of findings. IPA studies aim to illuminate lived experience through a sense‑making approach while preserving the particularity of individual participants. However, inherent methodological limitations include the inability to produce strictly reproducible findings or value‑neutral interpretations, given the researcher’s intentional immersion in and central role within the interpretive analytic process. As with most qualitative studies, our findings are also not intended to be generalizable to populations or to be able to make definitive claims of causality; rather, readers must assess the transferability of our insights to their own contexts. Interpretive work is also shaped by researcher positionality. Two of the authors are current MS2 and MS3 undergraduate medical students considering radiology, and they had strong rapport with interviewees. For IPA-based work, this can be seen as a strength, allowing the researcher to walk more closely along the journey described by participants. Mitigating the risk of bias, one member of our team is a PhD-trained medical educator with over 30 years of experience and currently serves as a student affairs dean with extensive background in career advising, who was able to seek justifications from an outsider perspective to ensure accountability in the primary analysis. We maintained a reflexive stance throughout data analysis to minimize personal biases and cross-checked each other’s work to ensure our interpretations were reasonable and defensible.

Upon conducting all interviews, it became apparent that pursuing radiology is a journey that varies from person to person. Many of our participants did not have an interest in radiology throughout most of medical school, with experiences on clinical rotations being key to ultimately exploring radiology. Nonetheless, many interviewees went through medical school set on pursuing an entirely different specialty and decided on radiology relatively late in medical school. Yet, for others, certain experiences and interests from their personal background essentially planted the seeds that led to their pursuit of radiology. Similar motivating factors for pursuing radiology as a career were seen across all three groups of interviewees. For example, all three groups appreciated the intellectual challenge of radiology and described a deep passion for learning. Furthermore, all mentioned the intersection of radiology with a variety of other specialties as having importance to them. Striking across all three groups of interviewees was the value interviewees placed on being able to drive patient care through radiology. This appears to be in line with another study that found that intellectual challenge and impact on patient care were two of the top three factors of importance for students applying to radiology [8]. Feeling a sense of competency and assurance in regard to personal skills and qualities that align with being in radiology was also of significance. 

The results of our interviews showed that it may take trial and error to find one’s ‘perfect fit’ in the field of medicine. In particular, the interviews showed that medical school is a period of time where motivating factors change. A prior study found that while there was no significant leading factor motivating specialty choice during the first year of medical school, interest in the field itself significantly increased in importance by the fourth year of medical school [5]. Many interviewees were able to identify interest in radiology and find meaning in pursuing the field only after exploring other specialties first. 

As noted, specialty interests can change significantly throughout medical school, as confirmed by studies showing that the positive predictive value of students’ initial specialty choices improves between the first and third years [13,14]. Several factors may contribute to this shift. Psychology research has identified four categories of professional motivation: prosocial, external, intrinsic, and loafing. One study suggests that medical students often begin their training with strong prosocial motivations, but high rates of burnout and loss of idealism may influence their eventual specialty decisions [10]. Other studies have emphasized interest in the specialty itself and income as major factors shaping specialty choice [5,15]. Our analysis showed that choosing radiology ultimately requires a deep understanding of one’s values, goals, and identity within medicine. Notably, our sample demonstrated strong indications of both intrinsic and prosocial motivation as key influences in the decision to pursue radiology. Additionally, participants consistently believed their personal qualities aligned well with the field, a finding worth considering, given that perceptions of competency have been shown to significantly impact specialty choice [16]. 

For the PGY-5 residents and radiologists, most expressed a sense of satisfaction with their ultimate career choice. While other studies have suggested that both radiology residents and radiologists experience burnout, our limited sample size may have impacted this theme [17,18]. Additionally, two of our radiologists had been in practice for over thirty years. Late-career physicians have been cited to be the most satisfied and have lower rates of distress [19].

Radiologists highlighted changes in the field they’ve observed throughout their career, including new technologies and new sub-specialties. As residents reflected on their training, they described how their appreciation for radiology grew upon being able to better understand it and its inherent complexities and nuances. Regarding perceptions and expectations of radiology, medical students and residents both commented on the training environment. Medical students expressed expectations for program-specific characteristics, while residents provided insight into the experience of radiology residency training. Interestingly, medical students’ expectations for radiology training aligned with the realities of those within the field. There were widespread agreement and confirmation among radiologists and residents that radiology is a highly academic field, requiring significant studying, dedication, and effort, despite common misconceptions. 

The radiologists, residents, and medical students interested in radiology all commented on the importance of addressing the misconceptions many medical students have about radiology in order to enhance interest in the field. While the interviewees in this study discussed the opportunities to make a significant impact on patient care in radiology at length, a previous study found that medical students going into radiology were much more likely to agree that radiologists play a vital role in diagnosis and patient management compared to students not going into radiology [8].

Through the interviews, all three groups expressed the desire for radiology to have a greater role in undergraduate medical education. Furthermore, many argued that mentorship is key in the pursuit of radiology. In fact, previous research has shown that residents who applied to radiology were more likely to have received positive mentorship [8]. The insights obtained in these interviews can help inform interventions aimed at motivating more students to pursue radiology as a career. Greater incorporation of radiology into formal medical school curricula, promoting earlier exposure to radiology, and providing opportunities for interaction with radiologists are key components to help provide medical students with a better understanding of radiology and consider the specialty. Requiring radiology rotations was a notable topic during interviews. A survey conducted by the American College of Radiology (ACR) found that students attending schools with required imaging rotations were more likely to pursue radiology [8]. However, only 16% of medical schools mandate such rotations [20].

**Figure 1:**
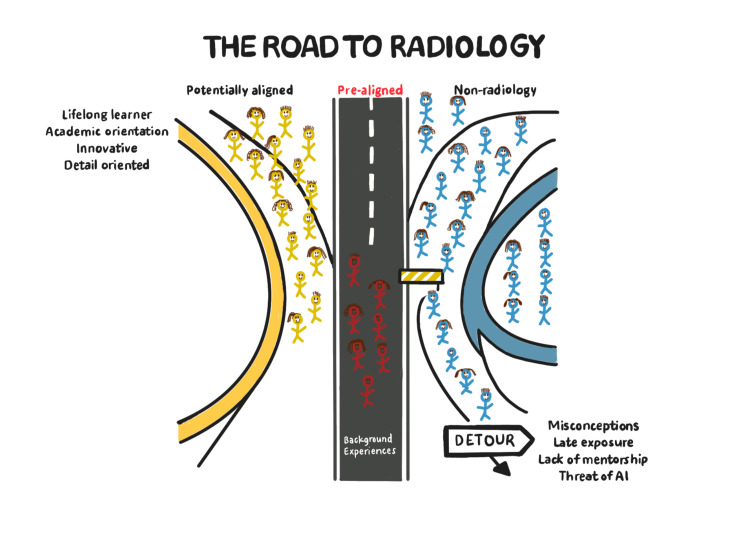
The Road to Radiology: A Conceptual Model Original figure by Sanjana Kumar.

From the interviews conducted in this study, we propose a conceptual model to explain how incoming medical students align with diagnostic radiology (Figure 1). A small group (“Pre-aligned”) enters medical school already considering radiology, shaped by prior experiences and interests. A larger group (“Potentially aligned”) may not initially prefer radiology but exhibits traits, such as curiosity, innovation, and attention to detail, linked to success in the field. They may eventually choose radiology or another specialty. The largest group (“Non-radiology”) is likely committed elsewhere but may be steered away from radiology by barriers like misconceptions, limited exposure, lack of mentorship, and concerns about AI. The framework highlights how personal predispositions and systemic factors shape specialty choice.

## Conclusions

These shared themes suggest that targeted interventions, particularly early integration of radiology into the medical curriculum and the provision of accessible mentorship, may foster greater interest in the field and help reduce barriers to entry. The findings offer actionable insights for medical educators seeking to support students in exploring radiology as a viable and fulfilling career option.
